# Enhancing Clinical Workflow Efficiency in Flow Cytometry Reporting with LLMs

**DOI:** 10.1007/s10875-026-02006-0

**Published:** 2026-03-24

**Authors:** Thomas E. Tavolara, Jake Meyer, Christopher Garcia, Attila Kumanovics, Patrick Vanderboom

**Affiliations:** Department of Laboratory Medicine and Pathology, Mayo Clinic, Rochester, USA

**Keywords:** Large Language Models (LLMs), Flow Cytometry Reporting, Immunophenotyping, Clinical Workflow Efficiency, Interpretive Reporting Automation, Primary Immunodeficiency Diagnostics

## Abstract

**Background:**

Accurate interpretation of clinical test results is essential for diagnosing and managing complex immunological disorders. We explored the potential of large language models (LLMs) to automate interpretative reports for immunodeficiency and immune competence assessment via quantitative lymphocyte profiling and B-cell subset phenotyping (QLP/BSP).

**Methods:**

An LLM was fine-tuned using parameter-efficient techniques on a dataset consisting of immunophenotyping test results and corresponding interpretive reports. Model performance was compared against a retrieval-based method and expert pathologist reports. A novel automated evaluation framework assessed both the accuracy of cell population comments and the clinical relevance of generated interpretations. A custom application was built to simulate clinical workflows and measure the impact on pathologist efficiency and accuracy.

**Results:**

Fine-tuned LLMs achieved accuracy comparable to expert pathologists in identifying and commenting on abnormal cell type counts and frequencies, in comparison the retrieval-based method exhibited substantial error rates. There was no significant difference between the rate at which abnormalities for cell subtypes were commented on between the LLMs and pathologists. More importantly, LLMs significantly reduced the time required for a single pathologist to finalize reports with a mean reduction in time of 29%.

**Conclusion:**

Our results suggest that LLMs hold promise for enhancing efficiency and consistency in the clinical laboratory setting. By automating aspects of interpretive reporting, LLMs can potentially reduce pathologist workload and improve the turnaround time for critical diagnostic information, while requiring expert pathologist oversight.

**Supplementary Information:**

The online version contains supplementary material available at 10.1007/s10875-026-02006-0.

## Introduction

Large language models (LLMs) have revolutionized the field of natural language processing (NLP), enabling tasks like translation, summarization, question answering, and text generation [1]. These models have gained significant attention and have been applied across numerous domains, most prominently healthcare and medicine [2]. Significant research investment (in parallel with general-purpose LLMs) has been made towards specialized LLMs like Med-PaLM and Med-PaLM2, which are designed to incorporate and apply domain-specific clinical knowledge [3, 4].

We propose applying LLMs to automatically generate preliminary interpretive reports for quantitative lymphocyte profiling and B-cell subset phenotyping by flow cytometry (QLP/BSP). QLP/BSP profiling is a critical analytical tool in immunology [5]. It provides valuable insights into the state of a patient’s immune system by characterizing and counting B-cell populations. Accurate interpretation of this test is essential for diagnosing and managing various immunological disorders [6]. At Mayo Clinic, the QLP/BSP profile consists of 34 individual test results that quantify the total CD45 + lymphocyte count and the relative (percentage) and absolute (cells/mcL) counts of the major lymphocyte subsets (T cells, B cells and NK cells), as well as 11 absolute and relative B cell subset counts (CD21+, CD21low, total memory B cells, non-switched memory, IgM-only memory, switched-memory, transitional, plasmablasts, total IgM+). Pathologists must manually review these results and write an interpretive report to summarize the test results to help with the diagnosis of common variable immunodeficiency or other B cell related disorders [7].

The validation of LLM responses is more complicated than typical machine learning applications, as the generated text contains multiple interdependent components (e.g., numeric values, categorical interpretations, and narrative explanations). However, automated metrics comparing generated text to a reference standard often fail to capture domain-specific meaning. For example, traditional metrics like BLEU [8] and ROUGE [9] are reflective of edit distance, measuring how much generated text overelaps with a reference standard. Slight variations, such as negation, aren’t penalized because they have a minor impact on textual overlap, even though they can substantially alter the semantic meaning of a sentence. BERTScore [10], a more sophisticated automated metric that measures generation accuracy, suffers less from such issues but has been shown not to correlate with human-preferred responses [11]. More recently, automated metrics leveraging LLMs to evaluate other LLMs have become popular [12]. However, subsequent studies on these tools have shown that they too can suffer from their own limitations [13, 14]. With ample resources, the gold standard for evaluating LLM outputs is reinforcement learning (i.e. iterative human-in-the-loop feedback). However, in applications requiring input from highly-trained experts (i.e. medicine), such a gold standard is practically limited. Therefore, scalable validation of highly-specialized LLM outputs (such as those in medicine) is still an outstanding problem. In this light, we developed an automated method to evaluate the accuracy of LLM-generated QLP/BSP interpretative reports without the need for medical expertise.

We evaluated the efficacy of fine-tuned LLMs in generating preliminary interpretive reports for QLP/BSP, comparing them to expert pathologist’s reports. Additionally, we evaluated a retrieval-based method (see Methods) that assembles reports from previously authored interpretations as a comparison to LLMs. The performance of our models was assessed using a novel automated evaluation framework that rigorously examined both the accuracy of cell population comments and the clinical relevance of the generated interpretations.

## Methods

**Fig. 1:**
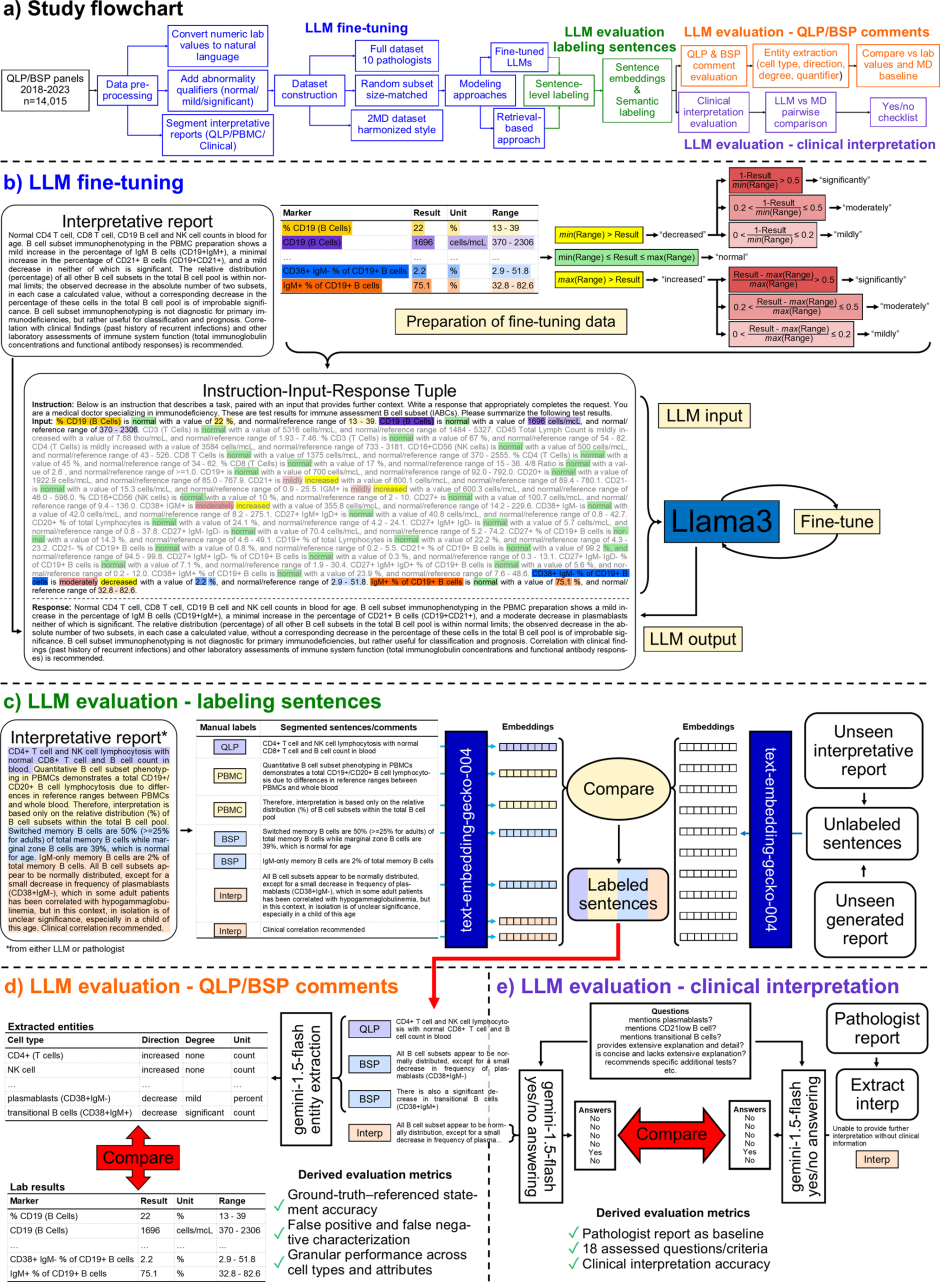
Study overview, model training, and automated evaluation framework. **(a)** QLP/BSP panels (2018–2023) were extracted and programmatically converted into natural-language representations, augmented with qualitative abnormality descriptors, and segmented into standardized interpretive sections. Three datasets were constructed to assess the effects of data size and reporting heterogeneity: a full dataset (10 pathologists), a size-matched random subset, and a harmonized two-pathologist (2MD) dataset. These datasets supported downstream model training and automated evaluation of QLP/BSP comments and clinical interpretations. **(b)** Example of a pathologist-authored interpretive report paired with its corresponding QLP/BSP laboratory values. Numeric test results are programmatically transformed into narrative descriptors (e.g., normal, mildly, moderately, or significantly increased/decreased), which are then assembled into instruction–input–response tuples for supervised fine-tuning. Color-coding highlights the correspondence between specific laboratory values, their qualitative descriptors, and the resulting text within the training examples. Instruction inputs are used to fine-tune the LLM, producing interpretive report outputs. **(c)** Illustration of the sentence labeling framework used to segment interpretive reports into QLP, PBMC, BSP, and clinical interpretation sections. Manually labeled sentences from reference reports are embedded using a text embedding model (text-embedding-gecko-004), generating label-aware semantic representations. Unseen (generated or held-out) reports are similarly embedded without labels, and sentence labels are assigned by comparing embeddings to the labeled reference space. **(d)** Sentences labeled as QLP or BSP comments are processed using an LLM-based entity extraction pipeline to identify referenced cell types, direction and degree of change, and whether abnormalities are expressed as counts, percentages, or both. Extracted entities are compared against raw laboratory values to establish a reference standard and quantify statement accuracy, false positives, false negatives, and granular performance across cell types and attributes. **(e)** Clinical interpretation sentences are evaluated using a structured, LLM-assisted comparison framework. Model-generated and pathologist-authored interpretations are independently assessed using binary (Yes/No) responses to a predefined set of clinically relevant questions. Responses are compared to derive evaluation metrics using the pathologist report as the reference standard, yielding quantitative measures of clinical interpretation fidelity across 18 assessment criteria

As illustrated in Fig. 1a, our Methods follow a sequential workflow spanning QLP/BSP dataset construction, LLM fine-tuning, sentence-level segmentation, and two complementary evaluation tracks assessing quantitative laboratory commentary and higher-level clinical interpretation.

### Datasets Description

The QLP/BSP panel comprises (i) whole-blood quantitative lymphocyte profiling (QLP) for T-, B-, and NK-cell enumeration and (ii) peripheral blood mononuclear cell (PBMC) based B-cell subset phenotyping (BSP). The combined panel includes 34 reportable results, including absolute counts and/or percentages for major lymphocyte populations (e.g., CD3, CD4, CD8, CD19, CD16/CD56) and 11 B-cell subsets (Table S1).

After Institutional Review Board (IRB) approval, all QLP/BSP panels available for secondary use and performed between 2018 and 2023 were extracted from Mayo Clinic’s laboratory information system (LIS). The dataset included 14,015 paired QLP/BSP panels with corresponding pathologist-authored interpretive reports, comprising 471,546 individual reported test results. Dataset-level and attribute-level characteristics are summarized in Table 1.

**Table 1:** Dataset characteristics and abnormality prevalence. Summary statistics describing report-level properties, contributing pathologists, report length, and the prevalence and distribution of abnormal findings across quantitative lymphocyte profiling (QLP) and B-cell subset phenotyping (BSP) components. Abnormality is defined as the presence of at least one laboratory value outside institutionally defined reference ranges. Confirmed diagnoses were not uniformly available and were therefore not used as labels

Characteristic	Value(s)
Number of reports	14,015
Number of pathologists	10
Median patient age (IQR)	37 (14–60)
Median report length in words (IQR)	120 (78–162)
Reports with ≥ 1 abnormal finding (%)	87
Median abnormal findings in reports with ≥ 1 abnormal finding (IQR)	7 (3–11)
Reports with ≥ 1 abnormal QLP finding (%)	57
Median abnormal QLP findings in reports with ≥ 1 abnormal finding (IQR)	3 (1–5)
Reports with abnormal PBMC (%)	38
Reports with ≥ 1 abnormal BSP finding (%)	81
Median abnormal BSP findings in reports with ≥ 1 abnormal finding (IQR)	6 (3–9)

Generally, interpretive reports are organized into up to four sections: (1) a QLP comment summarizing whole-blood T/B/NK findings; (2) a PBMC comment addressing lymphopenia/lymphocytosis when present; (3) a BSP comment describing B-cell subset abnormalities; and (4) a clinical interpretation section when indicated. A representative report with section labels is shown in Fig. 1c; additional preprocessing details are provided in the Supplementary Information (*Data pre-processing*).

### LLM Fine-tuning

To generate interpretive clinical laboratory reports, we fine-tuned a large language model (LLM) using fine-tuning. Fine-tuning refers to additional supervised training of an existing general-purpose language model on a smaller, domain-specific dataset so that its outputs align with a specific clinical task – here, narrative interpretation of QLP/BSP laboratory results. Training examples were formatted as instruction-input-response pairs to mirror the intended clinical use case (Fig. 1b). We fine-tuned the open-source Llama 3.1 (8-billion-parameter) model using a parameter-efficient approach, in which only a small subset of model parameters is updated while the pretrained weights remain fixed. This strategy substantially reduces computational requirements while preserving the model’s general language capabilities. After training, the adapted parameters were integrated with the base model for inference. Additional implementation details are provided in the Supplementary Information.

### LLM Evaluation – Labeling Sentences

To enable automated evaluation of narrative reports, we first developed a sentence-labeling framework to assign each sentence to its corresponding report section (QLP, PBMC, BSP, or clinical interpretation). A subset of interpretive reports from the training data (*n* = 1,120) was manually annotated at the sentence level to provide reference labels for this task.

Sentences were converted into numerical representations using Google’s text-embedding-gecko-004, which encodes text into vectors that capture semantic similarity (Fig. 1c). This embedding-based approach enabled automated sentence labeling across the remainder of the dataset. The same sentence-labeling pipeline was applied uniformly to both pathologist-authored reports and LLM-generated reports; no manual annotation was performed on generated outputs.

### LLM Evaluation – QLP, BSP, and PBMC Comments

**Fig. 2:**
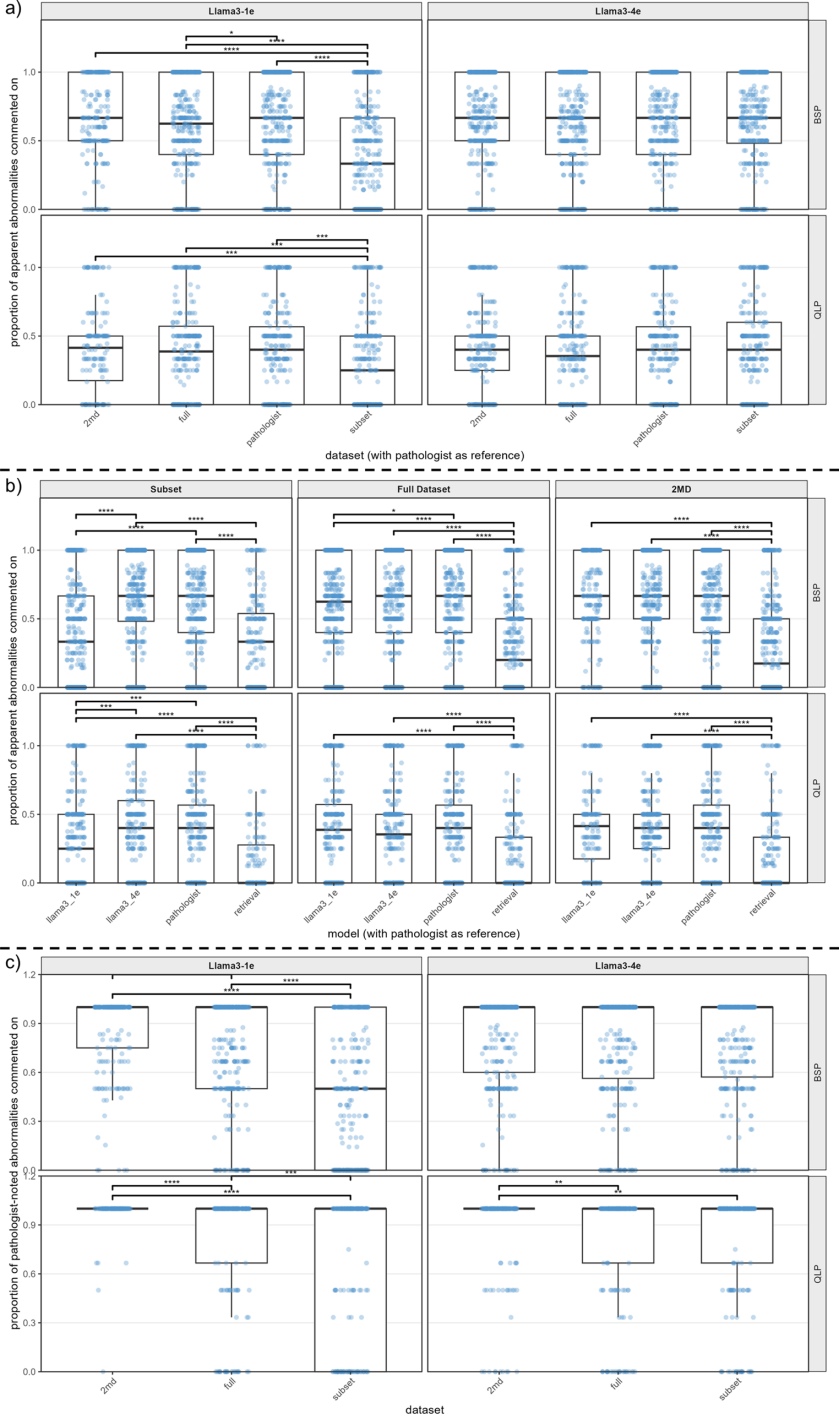
Proportion of cell count or cell percentage abnormalities commented on across methods (Llama3-4e, Llama3-1e, retrieval), datasets (full, subset, 2MD), and reference standards (lab results, pathologist). For example, commenting on 3 of 6 abnormalities yields a proportion of 0.5. Unpaired Wilcoxon tests were used to compare distributions. **a**) Compared with Llama3-1e, Llama3-4e comments on a larger proportion of abnormalities and does not differ significantly from pathologists across datasets. **b**) Across datasets, retrieval consistently underperforms, while increased data uniformity is associated with improved performance; Llama3-4e again does not differ significantly from pathologists. **c**) Using pathologists as the reference standard, greater data uniformity (2MD) further improves model performance

**Fig. 3:**
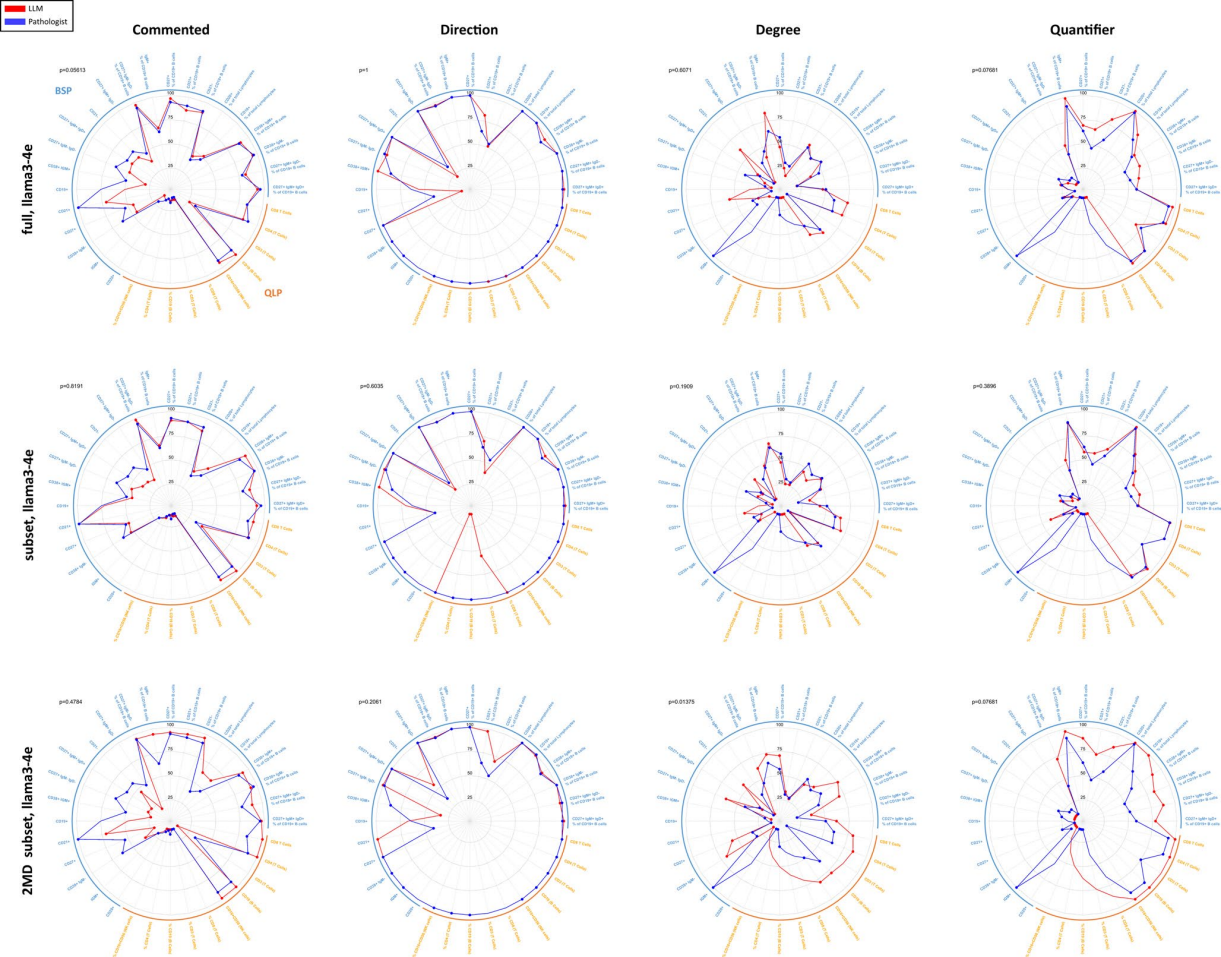
Direct comparison of comment rate and accuracy for Llama3-4e and pathologist across cell types. Each row represents a different expanded data partition. The first column (“commented”) shows the percentage of abnormal test results commented on. Of those commented on, columns 2–4 of each subfigure show the factuality of each comment in terms of direction, degree, and quantifier. Additional results corresponding to Llama3-1e can be found in Figure S1. *note that continuous curves include interpolated segments where metrics are undefined due to zero-denominator cases and that these interpolated regions should not be interpreted as measured performance values

**Fig. 4:**
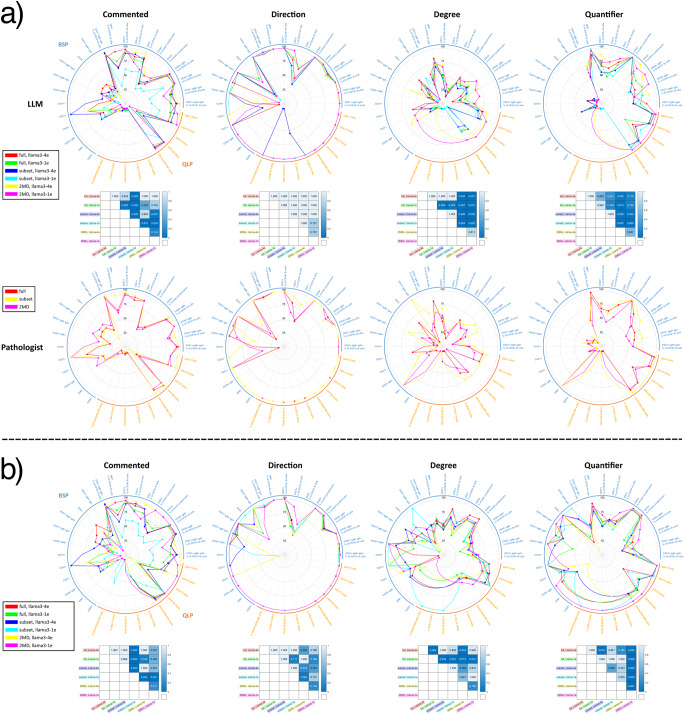
Abnormality commenting performance of LLMs versus pathologists across cell types. **a** Comparison of abnormality comment rate and accuracy LLM-based methods and pathologists across cell types. The first column (“commented”) shows the percentage of abnormal test results commented on. Of those commented on, columns 2-4 of each subfigure show the factuality of each comment in terms of direction, degree, and quantifier. Significant differences exist among all LLM/dataset combinations for comment rate, degree, and quantifier but not direction (*p* < 0.05, Friedman’s test). Pairwise comparisons (Wilcoxon signed-rank test, Benjamini-Hochberg corrected) reveal that comment rate for all model/dataset combinations is significantly greater than Llama3-1e/subset, Llama3-4e/subset is significantly greater than Llama3-1e/full, and Llama3-4e/subset is significantly greater than Llama3-1e/2MD. For degree and quantifier, 2MD-trained Llamas outperform full and subset trained Llamas. **b** Comparison of LLM-based methods normalized to pathologists across cell types. Proportion of abnormal tests commented on and accuracy in terms of direction, degree, and quantifier reported. The first column (“commented”) shows the percentage of abnormal test results commented on. Of those commented on, columns 2-4 show the factuality of each comment in terms of direction, degree, and quantifier. Each box plot is normalized to what the pathologist commented on rather than the raw lab values. Significant differences exist among all LLM/dataset combinations for comment rate, degree, direction, and quantifier (*p *< 0.05, Friedman’s test). Pairwise comparisons (Wilcoxon signed-rank test, Benjamini-Hochberg corrected) reveal that comment rate for all model/dataset combinations is significantly greater than Llama3-1e/subset, direction accuracy for Llama3-1e/subset is significantly greater than Llama3-4e/2MDs, degree accuracy for all model/dataset combinations is significantly greater than Llama3-1e/full, quantifier accuracy for Llama3-4e/subset is significantly greater than all model/dataset combinations (besides Llama3-4e/full), quantifier accuracy for Llama3-4e/full and Llama3-4e/subset is significantly greater than Llama3-1e/2MD.*note that continuous curves include interpolated segments where metrics are undefined due to zero-denominator cases and that these interpolated regions should not be interpreted as measured performance values

To quantitatively evaluate QLP and BSP narrative comments, each labeled sentence was processed to extract structured clinical attributes, including referenced cell types, direction of change (normal, increased, or decreased), degree of abnormality (mild, moderate, or significant), and quantifier (absolute count, percentage, or both). Attribute extraction was performed using Google’s Gemini Pro v1.5 in text-only mode; no multimodal inputs were used. Gemini Pro v1.5 was selected for ease of integration, and comparable LLMs could have been used for this task. Prompts used for extraction are provided in Table S2. Because equivalent concepts were often expressed using varied terminology, extracted entities were normalized to a standardized vocabulary using a manually curated alias dictionary (Tables S3-S6). All normalized entities were manually reviewed, and discrepancies were rare (*n* = 10, < 1%) and corrected. Extracted attributes were compared directly against the corresponding laboratory values, which served as the reference standard. This enabled identification of both unsupported statements (false positives) and omitted comments for abnormal results (false negatives) in both LLM-generated and pathologist-authored reports. Four complementary metrics were computed: comment rate, defined as the proportion of laboratory-defined abnormalities that are mentioned in the narrative report; direction accuracy, assessing whether the direction of abnormality (increased or decreased) was correctly described; degree accuracy, evaluating concordance between the reported and laboratory-defined magnitude of abnormality; and quantifier accuracy, assessing whether abnormalities were correctly described using counts, percentages, or both. Metrics were computed under two reference standards. In the primary analysis, abnormalities and attribute correctness were defined relative to raw laboratory values. In a secondary, normalized analysis, the same metrics were computed using pathologist-authored reports as the reference standard, with correctness defined by concordance with pathologist commentary. Metrics were summarized by QLP/BSP grouping, cell type, and attribute type using box plots and radar (spider) plots (Figs. 2, 3 and 4, Figure S1). Box plots depict the distribution of metric values across cell types, while radar plots provide a compact, multivariate summary of performance across all evaluated cell types, enabling direct visual comparison between models and the pathologist reference standard. See Supplementary Information for further details.

PBMC comments were evaluated separately. Because PBMC abnormalities are determined directly from specific laboratory values, the presence or absence of a PBMC comment was assessed via direct logical comparison to CD19 + and CD20 + counts.

### LLM Assessment – Clinical Interpretation

Clinical interpretation sections were evaluated using a structured framework designed to assess clinical concordance rather than surface-level textual similarity. Because interpretations may differ in phrasing while conveying equivalent meaning, we compared model-generated interpretations against pathologist-authored reports using a standardized set of binary clinical attributes.

Clinically relevant attributes were defined based on common elements present in expert interpretations (e.g., description of B-cell abnormalities, diagnostic considerations, and recommendations for further evaluation). Each attribute was recorded as present or absent within an interpretation. The same attribute checklist was applied uniformly to both LLM-generated and pathologist-authored reports.

For each attribute, agreement between model output and the pathologist reference was quantified using standard performance metrics, including accuracy, sensitivity, and specificity. This approach enables structured, clinically meaningful comparison of interpretive content beyond direct text similarity. Additional details, including the full attribute list and annotation procedures, are provided in the Supplementary Information.

### Retrieval-based Method

In addition to the LLM-based approach, we evaluated an unsupervised retrieval method based directly on laboratory values to predict QLP/BSP interpretative reports. Details can be found in *Retrieval-based method* in Supplementary Information.

### Experimental Design

The full dataset consisted of 14,015 samples; however, we also prepared two additional subsets of the full dataset (Table 2) to characterize the effect of sample size and pathologist report consistency on the LLM generated report accuracy. The “2MD” dataset consisted of reports written by two pathologists with harmonized interpretive report writing styles. To control for training data sample size, an additional subset of the “Full” dataset was prepared that included the same number of samples in the training data set relative to the “2MD” cohort. Both the full and random subset had 10 report writers.

**Table 2:** Datasets description

	Full	Subset	2MD
*n *(train)	13,515	7135	7135
*n* (test)	500	500	283

We applied three methods to each partition of the expanded dataset – Llama3 trained for 4 epochs, Llama3 trained for 1 epoch, and the retrieval-based method. We will refer to these as Llama3-4e, Llama3-1e, and retrieval for the remaining part of the manuscript.

### Clinical Validation and Timing Study

To assess efficiency of the LLM-generated report templates in a clinical setting, we developed a custom application to mimic the LIS used when pathologists interpret QLP/BSP panel results and compose interpretative reports. In the application, a toggle feature allowed a pathologist to work with a pre-populated interpretive report generated by one of the proposed methods. The application implemented a timing mechanism that captures the total amount of time taken to sign-out each interpretative report. After a round of training that included reviewing 5 QLP/BSP panels with and without LLM-generated report templates, a pathologist utilized the application to sign-out 20 QLP/BSP reports. Timings were statistically compared using a two-sample sign-rank test. Additionally, our physician rated the LLM-generated outputs on a 3-point Likert scale on four dimensions – helpfulness, hallucinations, omissions, and fluff. Hallucinations were defined as factually incorrect statements, omissions as important results left out, and fluff as benign statements that were not necessary.

## Results

### Comments on Abnormal Tests – LLMs and Pathologist Versus Lab Test as a Reference Standard

Llama3-1e resulted in a significantly lower abnormal result comment rate relative to the pathologist for both QLP and BSP results (Fig. 2a). Llama3-4e eliminated this difference. A similar trend was observed for models trained on the full dataset, though only for the BSP results (Fig. 2b). Restricting the dataset to two pathologists with a harmonized reporting style and controlling for dataset size, Llama3-1e and Llama3-4e were not affected by training length, as no significant difference was observed with respect to the pathologist. The performance of the retrieval-based method was poor regardless of the dataset used and was not considered in further experiments. Finally, a pairwise comparison of all Llama3-1e models illustrated that full and 2MDs dataset training outperformed subset training (Fig. 2b). As for Llama3-4e, no significant differences were observed among any models relative to the pathologist.

When results are broken down into individual test components (*n* = 34), we again observe that LLMs do not differ significantly from pathologist (Fig. 3, Figure S1). Specifically, the rate at which LLMs commented on specific cell type abnormalities did not differ significantly from that of pathologists (*p* > 0.05, Wilcoxon signed-rank test). Similar patterns in commenting between pathologists and Llama3-4e were also observed. For example, pathologists and Llama3-4e tended to comment on abnormalities in absolute counts for QLP, whereas relative percentages were more frequently commented for abnormalities in B cell subsets. This trend is expected, as low recovery of B cell subsets associated with PBMC isolation can lead to artifactually decreased absolute counts. Significant differences among varying LLMs and expanded dataset partitions (Fig. 4a). Comment rate, degree, and quantifier vary significantly across all LLM/dataset pairs (*p* < 0.05, Friedman’s test), but not direction. Pairwise tests (Wilcoxon, BH-corrected) show all combos beat Llama3-1e/subset on comment rate; Llama3-4e/subset also tops Llama3-1e/full and Llama3-1e/2MD. For degree and quantifier, 2MD-trained Llama3 outperform both full and subset-trained versions.

### Comments on Abnormal Tests – LLMs Versus Pathologist’s Report as a Reference Standard

Pathologists do not comment on every abnormality, as evidenced by the results of the previous section. Therefore, we normalized LLM metrics using pathologists’ reports as a reference standard (rather than lab test results). In this setting, we observed significant differences among methods (Fig. 2c). Again, grouping cell types by QLP/BSP, count, or frequency, we observe that uniformity in training data seems to improve performance, more data improves performance, and longer training time may or may not make a difference (Fig. 2c). However, unlike previous results, the difference is starker, illustrating that training on the 2MD dataset with Llama3-4e results in improved abnormal comment rate. When results are broken down by cell type, significant differences were found across all LLM/dataset combinations for comment rate, degree, direction, and quantifier (*p* < 0.05, Friedman’s test). Pairwise comparisons (Wilcoxon signed-rank test, Benjamini-Hochberg corrected) show that all models exceed Llama3-1e/subset in comment rate, while Llama3-1e/subset outperforms Llama3-4e/2MDs in direction accuracy. For degree, all models outperform Llama3-1e/full, and for quantifier, Llama3-4e/subset leads all combinations except Llama3-4e/full. Both Llama3-4e/full and subset also surpass Llama3-1e/2MD on quantifier accuracy (Fig. 4b). 

### Abnormal Comment Rate Depends on Prevalence of Abnormal Results

One somewhat contradictory observation was the lack of any visual difference between the non-normalized metrics (Fig. 4a) and normalized metrics (Fig. 4b). Each LLM/dataset combination comments on abnormalities at the same rate, regardless of reference standard. The same is true for direction accuracy, degree accuracy, and quantifier accuracy.

**Fig. 5:**
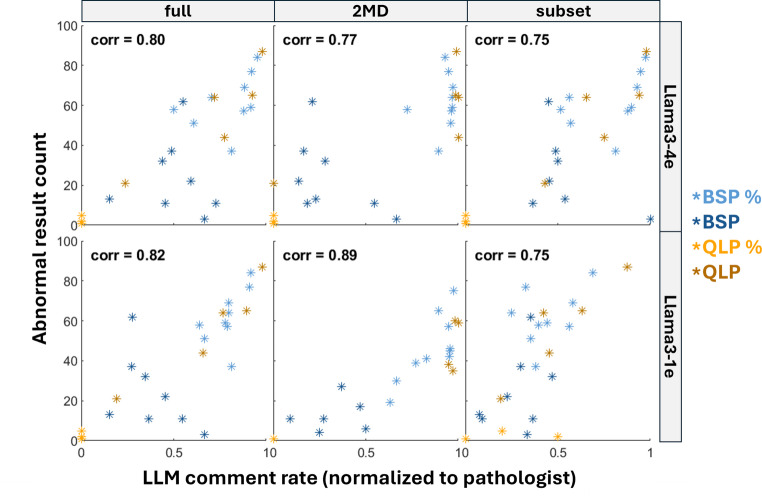
Correlation between LLM comment rate (normalized to pathologist) and abnormal test result count. Each star is a different cell type, colored by result type. Across all expanded dataset partitions, Llama3-1e consistently shows stronger correlations than Llama3-4e. BSP percentage and QLP count cell types cluster in the top right of plots, while QLP count and BSP percentage cluster in the bottom left

We also examined the correlation between abnormality prevalence and LLM comment rate (Fig. 5). Results indicate a strong correlation between all LLM/dataset combinations and abnormal result prevalence. The strongest correlation is seen for Llama3-1e/2MD, with a correlation of 0.8944. The weakest correlation is for Llama3-4e/subset, with a correlation of 0.7454. When comparing Llama3-4e to Llama3-1e across all dataset partitions, we observe a marked increase in correlation between abnormality frequency and comment rate for Llama3-1e. Simply training Llama for longer may result in less prevalence-dependent comment rate. We also observe that BSP percentage and QLP count cell types tend to cluster near the top right of each plot (i.e. high prevalence, high comment rate), while BSP count and QLP percentage cell types tend to cluster near the bottom left of each plot (i.e. low prevalence, low comment rate). This mirrors the results from all previous spider plots (Figs. 3 and 4, Figure S1).

### Incorrect Comments – LLMs and Pathologist Versus Lab Tests as a Reference Standard

Both LLMs and pathologists make qualitative cell type statements that are not supported by raw test results (Table 3). Again, as observed in previous results, uniformity in training data improves performance for LLMs, longer training improves performance for LLMs, more data improves performance for LLMs, more data does not improve retrieval, and uniformity in training data decreases performance for retrieval. We also observe that for Llama3-4e/full and Llama3-4e/subset, the error rate is similar to pathologists. Overall, retrieval performs very poorly relative to pathologists. When looking at specific cell types, some of the most common errors are statements regarding total memory B cells, for both pathologists and LLM methods (Figure S2). We also observe a high error rate for comments on non-switched memory B cells and NK cells for the 2MD dataset. Finally, we notice the error rate for pathologists on the 2MD dataset is much smaller (1.0%) than on the full dataset (3.8%), suggesting some variability across pathologists when writing interpretative reports.

**Table 3:** Error rates for LLMs and retrieval with pathologists as reference

		Pathologist	Llama3-4e	Llama3-1e	Retrieval
All	*Full*	3.8% (51/1355)	3.4% (43/1283)	5.5% (70/1264)	69.9% (504/721)
	*Subset*	3.8% (51/1355)	3.8% (52/1351)	12.0% (101/839)	69.6% (507/728)
	*2MD*	1.0% (8/803)	3.1% (42/1350)	5.7% (43/753)	77.6% (315/406)
QLP %	*Full*	0.0% (0/1355)	0.0% (0/1283)	0.0% (0/1264)	2.2% (16/728)
	*Subset*	0.0% (0/1355)	0.0% (0/1351)	0.4% (3/839)	2.5% (10/406)
	*2MD*	0.0% (0/803)	0.1% (1/1350)	0.3% (2/753)	1.4% (10/721)
QLP	*Full*	0.2% (3/1355)	1.1% (14/1283)	1.4% (18/1264)	26.1% (190/728)
	*Subset*	0.2% (3/1355)	1.2% (16/1351)	4.6% (39/839)	41.9% (170/406)
	*2MD*	0.2% (2/803)	0.6% (8/1350)	2.9% (22/753)	26.8% (193/721)
BSP %	*Full*	0.9% (12/1355)	1.2% (16/1283)	1.6% (20/1264)	11.1% (81/728)
	*Subset*	0.9% (12/1355)	1.4% (19/1351)	2.3% (19/839)	12.8% (52/406)
	*2MD*	0.0% (0/803)	0.2% (3/1350)	0.5% (4/753)	14.0% (101/721)
BSP	*Full*	0.1% (1/1355)	0.0% (0/1283)	0.1% (1/1264)	11.7% (85/728)
	*Subset*	0.1% (1/1355)	0.1% (2/1351)	0.5% (4/839)	18.5% (75/406)
	*2MD*	0.0% (0/803)	0.1% (1/1350)	0.3% (2/753)	13.6% (98/721)
PBMC comment	*Full*	0.0% (0/60)	1.5% (1/66)	4.1% (2/49)	1.5% (1/67)
	*Subset*	0.0% (0/60)	0.0% (0/69)	4.6% (3/65)	0.0% (0/62)
	*2MD*	0.0% (0/17)	0.0% (0/25)	0.0% (0/17)	7.7% (1/13)

### Clinical Interpretation

**Fig. 6:**
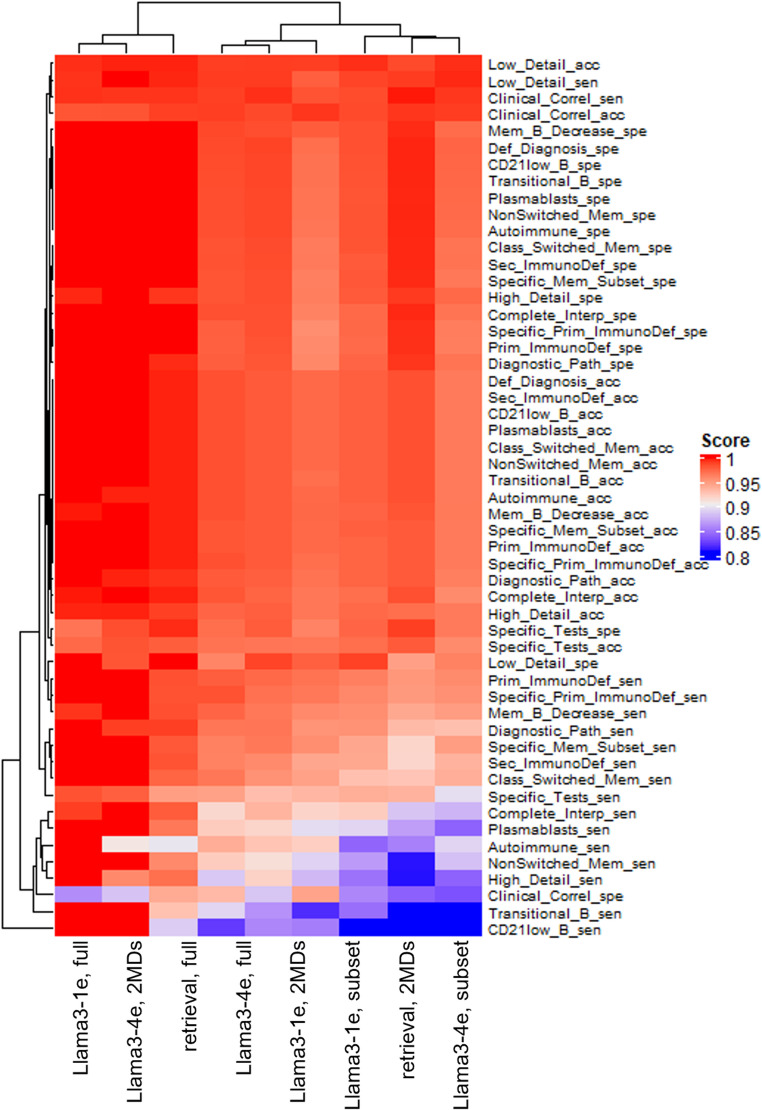
Heatmap of binary clinical interpretation metrics comparing generated reports to pathologist-authored references. Each cell represents the proportion of correct matches (Yes/No) between generated and reference reports for a specific clinical feature. ‘acc’ refers to accuracy, ‘spe’ to specificity, and ‘sen’ to sensitivity. Higher values indicate closer alignment with the reference standard (i.e. pathologists). LLM-based approaches outperform the retrieval-based method across nearly all categories. Among LLMs, Llama3-4e/2MD achieves the highest overall fidelity to pathologist interpretations, particularly in clinically specific and mechanistic features

Figure 6 presents a heatmap summarizing the performance of each method across clinical interpretation metrics. The values in the heatmap represent the proportion of matches between generated and reference interpretations for each clinical feature. Overall, LLM-based methods substantially outperformed the retrieval-based method. Retrieval methods often captured general patterns or surface-level phrasing but failed to reproduce the level of clinical nuance and specificity found in pathologist-authored reports.

Among LLMs, Llama3-4e/2MD consistently achieved the highest agreement with reference standard interpretations across the majority of binary categories. Notably, it excelled in sensitivity to relevant metrics. These results suggest that fine-tuned LLMs are capable of generating not only semantically aligned reports, but also clinically coherent and actionable interpretations that align closely with expert reasoning. This highlights their potential as decision support tools in the context of laboratory medicine and interpretive reporting.

### Timing Study and Qualitative Assessment by One Pathologist

**Fig. 7:**
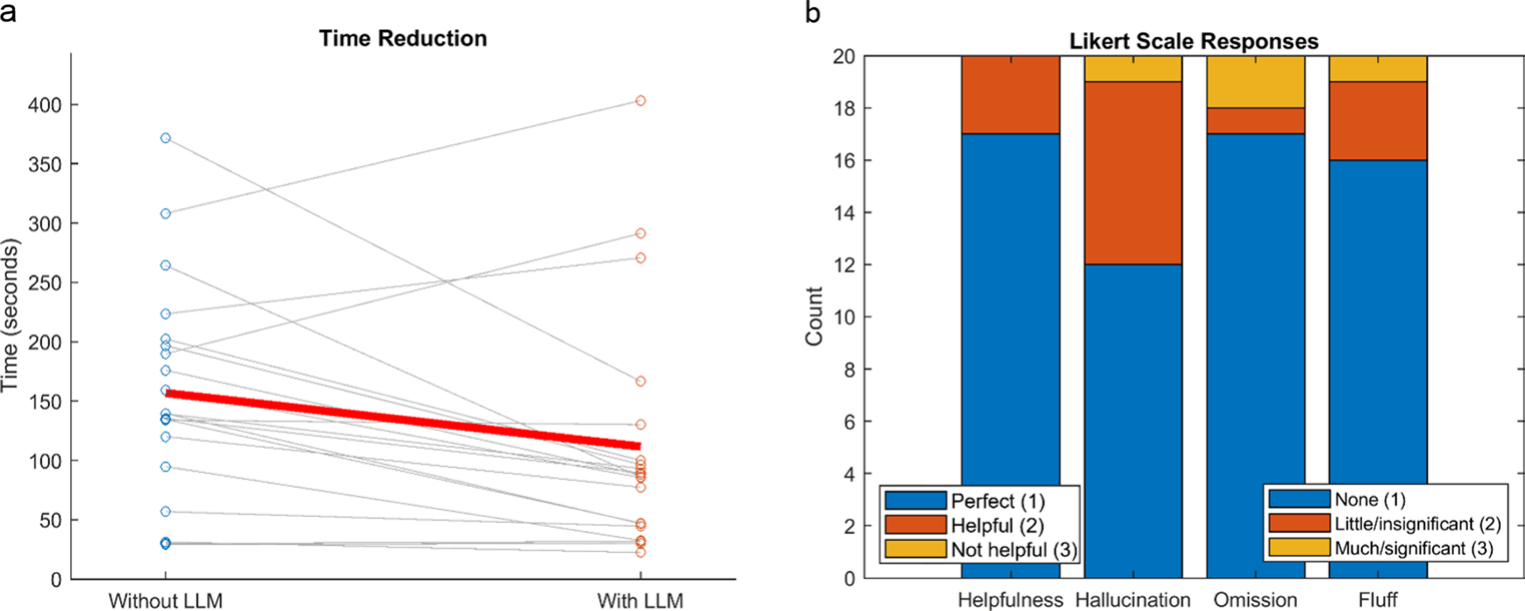
Quantitative and qualitative assessment by pathologist. (**a**) Timing analysis of 20 Llama3-4e/2MD test cases showed a significant reduction in interpretation time using LLM-generated reports (*p* = 0.0120), with a mean decrease of 29% (median 38%); 40% of cases saw > 50% time savings, though 3 cases took longer. (**b**) Qualitative assessment showed high satisfaction: 17 reports rated as perfect and 3 as helpful – matching those with longer times. Minor hallucinations were noted in 7 reports, one with major hallucinations. Omissions and fluff were mostly minimal

To assess generated report quality and efficiency gains in a clinical setting, one pathologist performed a timing and qualitative assessment on 20 test cases from Llama3-4e/2MD (Fig. 7a). Results indicate a significant decrease in time for interpretative reports when starting with LLM-generated reports (*p* = 0.0120, sign-rank). We also observed a mean reduction in time of 29% (median of 38%), with 40% of cases resulting in more than 50% reduction in time. Three cases resulted in significant increases in interpretation time. There was no significant difference between the first ten reviewed and second ten reviewed, both without LLM (*p* = 0.4316, sign-rank) and with LLM (*p* = 0.3223, sign-rank).

As for qualitative assessments, one pathologist was overall satisfied with LLM-generated reports (Fig. 7b). On the helpfulness scale, 17/20 LLM-generated reports were rated as perfect, while the remaining three were rated helpful. These were the same as the three that resulted in longer interpretation times. Our pathologist also noted 7 reports had little/insignificant hallucinations, while one had significant hallucinations. As for omissions, one had little/insignificant, while two had much/significant. Finally, three had little extraneous information (fluff), while one had significant fluff.

## Discussion

This study investigated the potential of fine-tuned LLMs to automate aspects of clinical laboratory interpretive reporting. Llama3-4e fine-tuned on the specialized 2MD dataset achieved performance comparable to expert pathologists in identifying and commenting on abnormal cell type counts and frequencies, with cell-type–specific differences that may inform staged deployment. We also observed a strong correlation between abnormality prevalence and LLM comment rate, highlighting a vulnerability in detecting rare conditions. In a clinical simulation, Llama3-4e/2MD generated clinically relevant interpretations and reduced the time required for one pathologist to finalize reports, supporting the role of LLMs as preliminary report writers rather than autonomous interpreters. In this assistive role, the model primarily enhanced workflow by consistently summarizing common abnormalities, organizing content into familiar interpretive sections, and reducing the clerical burden of composing repetitive narrative text. Conversely, reliability decreased for rare abnormalities and nuanced clinical judgment, reinforcing the need for expert oversight prior to sign-out.

We evaluated model-generated interpretative sections using binary clinical attributes derived from pathologist reports and computed accuracy, sensitivity, and specificity for each attribute (Fig. 6). Sensitivity most clearly differentiated modeling approaches, whereas specificity was similar across configurations. This pattern suggests that models were comparably conservative in avoiding pathologist-absent elements but differed in how consistently they included expected interpretive elements, an effect most favorable to Llama3-4e/2MD. Framing performance in these terms helps clarify which interpretive components are captured reliably and which remain prone to omission and therefore warrant targeted safeguards.

The strongest performance occurred when fine-tuning was restricted to semantically similar reports authored by two pathologists, indicating that reduced linguistic and interpretive variability improves reliability. From a production perspective, this should be interpreted less as reliance on individual authors and more as evidence that constraining stylistic and semantic degrees of freedom is important for safe deployment. In multi-pathologist environments, this can be supported by standardized reporting templates, controlled model scope (e.g., limiting output to well-defined interpretive domains), and modular or tiered fine-tuning aligned with group-level consensus rather than individual practice patterns.

These findings support implementation and deployment as a human-in-the-loop tool: generate a draft at result availability, then review and edit before sign-out. Refinement strategies include targeted augmentation for rare abnormal patterns, rule-based checks to improve data grounding, and confidence-aware signaling to highlight cases requiring heightened scrutiny.

Several limitations warrant emphasis. The prevalence/comment-rate relationship likely reflects limited exposure to low-frequency abnormalities during training, analogous to class imbalance, and suggests that rare-condition sensitivity remains a key challenge. Our error analysis also showed that both LLMs (and, at times, pathologist-authored narratives) can contain statements not supported by the available laboratory values, underscoring the importance of data grounding; some apparent discrepancies may reflect additional clinical context available at the time of interpretation. In addition, qualitative review identified hallucinations and omissions in LLM-generated reports [15], which may carry higher risk in complex or low-prevalence cases. The clinical simulation was limited to 20 cases evaluated by a single pathologist, and three cases required more time with Llama3-4e than without, largely due to case complexity; time savings may not generalize across pathologists, workflows, or institutions.

Future work should prioritize broader and more diverse training data, strategies to improve rare-abnormality performance, and methods that explicitly detect errors and hallucinations rather than relying solely on improved data quality or newer models [16]. We also lacked a practical metric to predict when an LLM draft will meaningfully reduce interpretation time; future studies should evaluate utility-oriented measures that better correlate with time or cost savings.

In conclusion, fine-tuned LLMs can generate clinically useful draft interpretations for QLP/BSP and may improve efficiency and consistency when used with expert oversight. Remaining challenges, particularly rare-abnormality sensitivity and data grounding, support a development path centered on error detection, quantitative assessment of clinical benefit, and case-level uncertainty estimation to guide selective escalation or suppression of LLM-generated drafts.

## Supplementary Information

Below is the link to the electronic supplementary material.

Supplementary Material 1 (DOCX 10.3 MB)

## Data Availability

No datasets were generated or analysed during the current study.
