# Supplementary material for: Enhancing Clinical Workflow Efficiency in Flow Cytometry Reporting with LLMs
Source: J Clin Immunol. 2026 Mar 24;46(1):40. doi: 10.1007/s10875-026-02006-0 (PMC13053331; doi:10.1007/s10875-026-02006-0)
Supplement: Supplementary file 1 — Supplementary Material 1 (DOCX 10.3 MB) [file 10875_2026_2006_MOESM1_ESM.docx]

**Supplementary Materials**

**METHODS**

**Data pre-processing**

Given that LLMs are constructed to understand and generate natural language, all 34 numeric absolute and relative lymphocyte counts from the QLP/BSP panel were programmatically converted into a natural language narrative format. Additionally, for each reportable result, qualifying adjectives were added to the narrative with the aim of improving the LLMs ability to contextualize the magnitude of change relative to each analytes normal reference interval (RI). For example, if a result was less than 20% above or below the RI, the qualifying adjective "mildly" was added. If between 20% and 50% above or below the RI, the qualifying adjective "moderately" was added. If more than 50%, "significantly" was added. If the test result was normal, then a single qualifying adjective "normal" was added.

**LLM fine-tuning**

To generate interpretive clinical laboratory test reports, we fine-tuned the Llama 3.1 8B large language model using a supervised fine-tuning approach. Fine-tuning refers to adapting a pretrained language model using task-specific examples so that it performs a specialized function, rather than retraining the model from scratch. The Llama 3.1 model was selected for its open-source accessibility and strong performance in natural language generation.

We employed QLoRA (Quantization-aware Low-Rank Adaptation), a memory-efficient fine-tuning method that combines model weight compression with selective parameter updating. In this approach, the base model weights are stored in low numerical precision (4-bit quantization) to reduce memory usage, while small trainable adapter layers are added to the model. This enables effective fine-tuning while modifying fewer than 1% of the model’s parameters and operating within limited computational resources. The fine-tuning data were organized using the ShareGPT schema, a widely used conversational data format in which each training example consists of a user prompt and an assistant response. This structure mirrors real-world interactions with language models and supports supervised learning. Inputs and outputs were further formatted using the ChatML template, a standardized markup format that explicitly labels system instructions, user input, and model responses to ensure consistent interpretation during training. Training was conducted using the Unsloth framework (https://github.com/unslothai/unsloth), which provides optimized memory management and accelerated training on single-GPU systems. Model optimization was performed using AdamW, a commonly used optimization algorithm for neural networks that adjusts model parameters during training while applying weight decay to reduce overfitting and improve generalization.

Parameter-efficient fine-tuning was implemented via Low-Rank Adaptation (LoRA), which introduces small, trainable low-rank matrices into selected layers of the model while keeping the original pretrained weights fixed. This allows the model to learn task-specific behavior with minimal parameter updates. We additionally employed rank-stabilized LoRA (rSLoRA), an extension of LoRA designed to improve training stability and performance when adapting large models under constrained precision and memory settings. Fine-tuning was performed on the “unsloth/Meta-Llama-3.1-8B-bnb-4bit” architecture using a maximum sequence length of 2048 tokens. Training utilized a learning rate of 3 × 10⁻⁴ with a linear learning rate scheduler and a brief warmup phase. Gradient checkpointing was enabled to further reduce memory usage. Training was conducted on a single NVIDIA T4 GPU (16 GB VRAM) and completed in approximately two hours.

After training, the LoRA adapter weights were merged with the base model using 16-bit precision to create a standalone model for deployment. The resulting fine-tuned model demonstrated improved ability to generate accurate, contextually appropriate interpretive clinical laboratory reports.

**LLM evaluation – QLP, BSP, and PBMC comments**

Radar (spider) plots were used to visualize performance across multiple related metrics simultaneously. In these plots, each axis corresponds to a distinct QLP or BSP cell type, with values increasing radially from the center. Performance across all cell types is represented as a closed polygon formed by connecting values along each axis. Larger polygonal area reflects stronger overall performance across metrics, while deviations along individual axes highlight strengths or weaknesses for specific cell types or attributes. Overlaying polygons for multiple models enables direct visual comparison against the pathologist-authored reference standard; greater overlap indicates closer agreement. This visualization approach is particularly useful in this study, where 32 interrelated metrics must be interpreted jointly rather than independently. Note that continuous curves include interpolated segments where metrics are undefined due to zero-denominator cases and that these interpolated regions should not be interpreted as measured performance values

**Clinical interpretation assessment**

1. *Generation of pairwise comparative summaries*

For each pair of predicted and reference standard clinical interpretations, gemini-1.5-flash was prompted to produce a paragraph summarizing the semantic and clinical similarities and differences. The prompt used was “Summarize in one paragraph the similarities and differences (in terms of semantic similarity and clinical similarity) between the two following interpretations.”

1. *Induction of evaluation categories*

Gemini-1.5-flash was provided with a representative sample of these comparative summaries and prompted to extract common categories of similarities and differences observed across examples. The prompt used was “These are a summary of similarities and differences between two clinical interpretations. Can you please create categories of similarities and differences that robustly capture the variation seen in these examples?” The generated categories were manually reviewed and edited for clarity and accuracy. The final set of categories included both commonalities (e.g., universal recommendation for clinical correlation) and areas of variation (e.g., specificity of recommended next steps, detail of immunological explanation, mention of diagnostic limitations, etc.).

1. *Conversion to binary evaluation schema*

To enable structured analysis, gemini-1.5-flash was prompted to convert the qualitative categories into binary (Yes/No) metrics. The prompt used was “Can you please break down these characteristics into binary categories? You may split one characteristic into many if appropriate.” The result was a standardized checklist of binary features capturing the presence or absence of specific attributes in the interpretations. These included whether the interpretation…

explicitly mentions a decrease in memory B cells (Yes/No)

specifies a particular memory B cell subset (e.g., marginal zone, class-switched) (Yes/No)

specifically mentions class-switched memory B cells (Yes/No)

specifically mentions non-switched memory B cells (or marginal zone B cells).

mentions plasmablasts (Yes/No)

mentions CD21low B cells (Yes/No)

mentions transitional B cells (Yes/No)

mentions primary immunodeficiency as a potential cause (Yes/No)

names a specific primary immunodeficiency (e.g., CVID) (Yes/No)

mentions secondary immunodeficiency (e.g., immunosuppression, allo-HSCT) as a potential cause (Yes/No)

mentions autoimmune disease as a potential cause (Yes/No)

offers a specific diagnosis (e.g., CVID) (Yes/No)

suggests specific further tests or a clear diagnostic pathway (Yes/No)

explicitly recommends correlating the lab results with clinical findings (Yes/No)

provides extensive explanation and detail (Yes/No)

is concise and lacks extensive explanation (Yes/No)

recommends specific additional tests (Yes/No)

offers a relatively complete assessment based on the available data (Yes/No)

1. *Application of binary evaluation criteria*

Finally, each generated interpretation and its corresponding reference standard (i.e. the pathologist-written report) were re-evaluated using the binary criteria developed in Step 3. For each criterion, gemini-1.5-flash was prompted to answer with a binary response (“Yes” or “No”), comparing the generated interpretation to the reference. The prompt used was: “This is an interpretation for immune assessment of B cell subsets. Please answer only 'Yes' or 'No' for the following question with respect to the clinical interpretation.” Responses were manually reviewed and corrected when necessary to ensure consistency and accuracy.

1. *Final metric construction*

Each interpretation pair was thus scored across a consistent set of binary clinical attributes, enabling quantitative comparison. These structured metrics allowed us to assess the degree to which generated interpretations captured clinically relevant elements found in pathologist reports, beyond surface-level textual similarity.

For each binary clinical attribute (e.g., mention of class-switched memory B cells, recommendation for clinical correlation), model-generated interpretations were compared directly to the corresponding pathologist-authored reports, which served as the clinical reference standard. This comparison yielded a 2×2 confusion matrix for each attribute, consisting of true positives (attribute present in both model output and reference), false positives (present in model output but absent in reference), false negatives (absent in model output but present in reference), and true negatives (absent in both). From these confusion matrices, we derived standard classification metrics: accuracy, defined as the proportion of correct classifications across all cases; sensitivity, defined as the proportion of reference-positive cases correctly identified by the model; and specificity, defined as the proportion of reference-negative cases correctly identified.

**Retrieval-based method**

The retrieval-based method consisted of three steps. First, quantitative lab values were converted into qualitative categories (increased, decreased, normal) based on proximity to each component’s respective reference interval. Subsets of test results were then manually associated with each comment type – in Table S1, QLP for the QLP comment (e.g. those component tests performed on whole blood), CD19+ and/or CD20+ for the PBMC comment (i.e. those component tests corresponding to B cell counts), BSP for BSP cell comments (i.e. those component test corresponding to B cell subsets), and finally all rows for clinical interpretation. Finally, each lab value was converted to a z-score. This mean and standard deviation for z-score were derived from the reference range by taking the midpoint of the reference range as the mean and the lower and upper bounds of the reference range as ±3 standard deviations. This resulted in a vector of z-scores for each comment type.

Retrieval was performed on a comment-basis (i.e. whole QLP, PMBC, BSP, interpretative section, not one sentence) in two steps. First, the set of comments with the maximum number qualitative lab test matches (i.e. matches between increased/decreased/normal) are retrieved. Second, among these retrieved comments, the comment nearest to the query via Euclidean distance computed with respect to query and retrieval z-score vectors (i.e. is selected. Accuracy assessment of the retrieval-based method is carried out using the same method outlined above for the LLM generated and pathologist written reports.

|  | Figure S1. Direct comparison of comment rate and accuracy for LLM-based methods and pathologist across cell types. Each row represents a different LLM and expanded data partition combination. The first column (“commented”) shows the percentage of abnormal test results commented on. Of those commented on, columns 2-4 of each subfigure show the factuality of each comment in terms of direction, degree, and quantifier. p-values are based off a Wilcoxon signed-rank test. Only Llama3-1e/subset comment rate, Llama3-4e/2MD degree accuracy resulted in significant differences. |
| --- | --- |

| 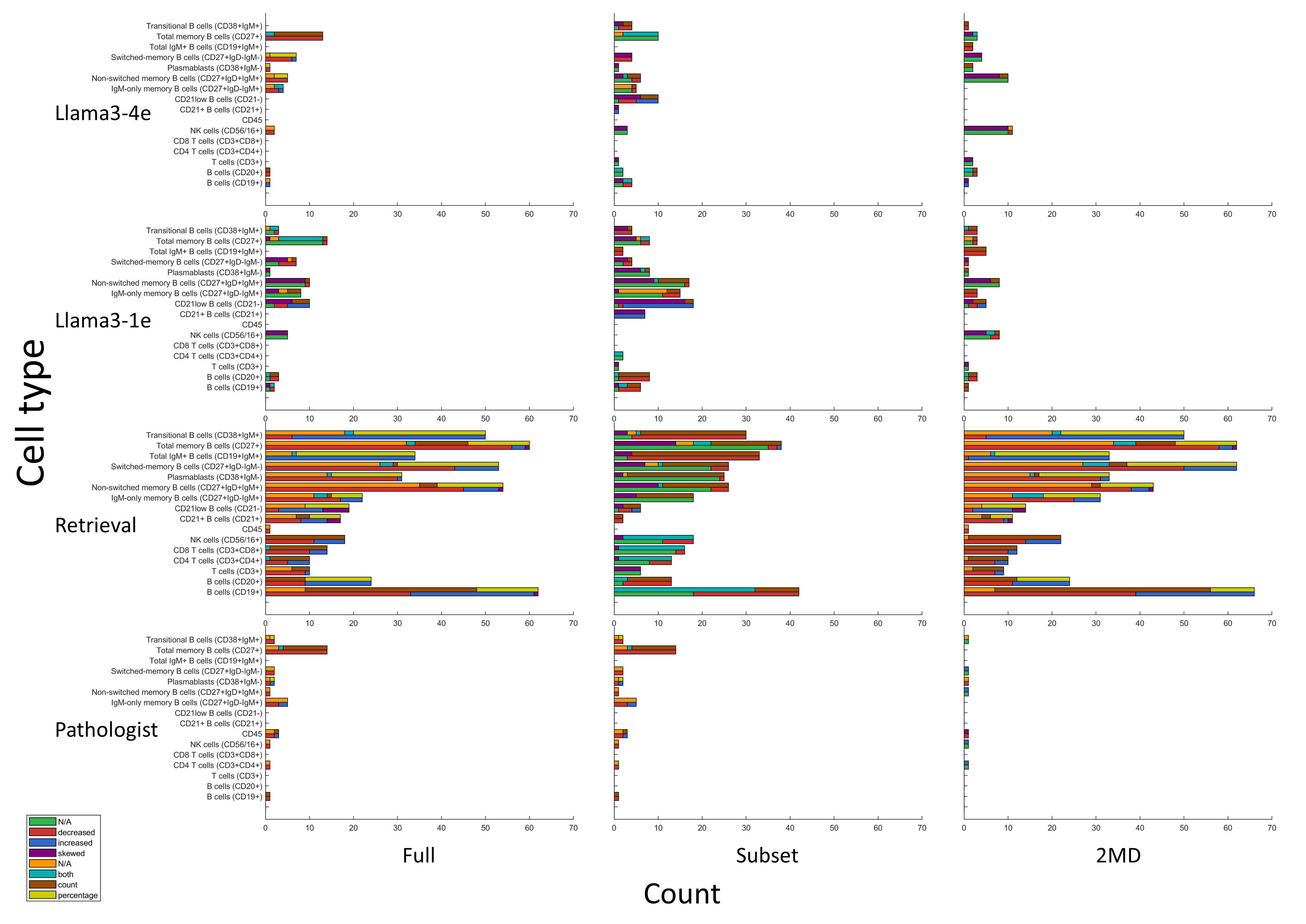 |
| --- |
| Figure S2. Error rates for comments about specific cell types among various experimental conditions. Among specific cell types, total memory B cells are a common source of error for both pathologists and LLMs. High error rates also appear in comments on non-switched memory B cells and NK cells within the 2MD dataset. Notably, pathologist error on the 2MD dataset is much lower (1.0%) compared to the full dataset (3.8%), suggesting variability in interpretive reporting across different cases or individuals. |

Table S1. Overview of the QLP/BSP Panel: Cell Populations, Sample Sources, Markers, and Reported Results

| **Panel Component** | **Cell Population / Subset** | **Defining Immunophenotype** | **Specimen Type** | **Percent Reported As** | **Absolute Count (cells/µL)** |
| --- | --- | --- | --- | --- | --- |
| **QLP (Whole Blood)** | Total lymphocytes | CD45+ (lymphocyte gate) | Whole blood | % of total leukocytes | Yes |
|  | Total T cells | CD3+ | Whole blood | % of CD45+ lymphocytes | Yes |
|  | CD4 T cells | CD3+ CD4+ | Whole blood | % of CD45+ lymphocytes | Yes |
|  | CD8 T cells | CD3+ CD8+ | Whole blood | % of CD45+ lymphocytes | Yes |
|  | CD4:CD8 ratio | CD3+ CD4+ : CD3+ CD8+ | Whole blood | - | Derived |
|  | Total B cells | CD19+ | Whole blood | % of CD45+ lymphocytes | Yes |
|  | Natural killer (NK) cells | CD16+ and/or CD56+, CD3− | Whole blood | % of CD45+ lymphocytes | Yes |
| **BSP (PBMC-based)** | Total B cells (reference) | CD19+ | PBMCs | % of CD19+ B cells | Derived* |
|  | Total memory B cells | CD19+ CD27+ | PBMCs | % of CD19+ B cells | Yes† |
|  | Marginal zone / non-switched memory B cells | CD19+ CD27+ IgD+ IgM+ | PBMCs | % of CD19+ B cells | Yes† |
|  | IgM-only memory B cells | CD19+ CD27+ IgD− IgM+ | PBMCs | % of CD19+ B cells | Yes† |
|  | Class-switched memory B cells | CD19+ CD27+ IgD− IgM− | PBMCs | % of CD19+ B cells | Yes† |
|  | IgM B cells | CD19+ IgM+ | PBMCs | % of CD19+ B cells | Yes† |
|  | Transitional B cells | CD19+ CD38+ IgM+ | PBMCs | % of CD19+ B cells | Yes† |
|  | Plasmablasts | CD19+ CD38+ IgM− | PBMCs | % of CD19+ B cells | Yes† |
|  | CD21-low B cells | CD19+ CD21− | PBMCs | % of CD19+ B cells | Yes† |
|  | Mature B cells | CD19+ CD21+ | PBMCs | % of CD19+ B cells | Yes† |
|  | CD19/CD20 coexpressing B cells | CD19+ CD20+ | PBMCs / WB | % of CD45+ lymphocytes | Yes |

†Absolute counts for B-cell subsets are reported in cells/µL but are not directly measured in PBMCs.

Absolute counts for BSP subsets are derived by applying PBMC-based subset percentages to the whole-blood CD19+ absolute count obtained from QLP.

QLP measurements are performed using a single-platform, bead-based, 7-color lyse–no-wash flow cytometry assay.

BSP measurements use Ficoll-isolated PBMCs and a multicolor, multi-tube flow cytometry panel.

Percentages for QLP populations are relative to CD45+ lymphocytes, whereas BSP subset percentages are relative to CD19+ B cells, unless otherwise noted.

Age-adjusted reference ranges are provided in the clinical report.

Table S2. The extraction prompt for cell types, their change of direction, degree of change, and kind of change. This template was paired with every pathologist report and generated WB or B cell comment.

| Extract the cell types out of this sentence as they appear in the text. Extract whether there is a change resulting in increased, decreased, skewed, or neither. If increased or decreased, extract whether the degree of change is mild ('mild', 'small', 'minimally', 'mildly', 'Mild', 'Small', 'Minimally', 'Mildly'), moderate ('moderate', 'modest', 'Moderate', 'Modest'), or significant ('significant', 'marked', 'Significant', 'Marked'). If increased or decreased, extract if the change type is percentage ('percentage',' frequency'), count ('count', 'counts', 'absolute'), both, or neither.  If no change is mentioned and if the cell type is of total memory B cells, extract the associated value (%).  Respond in a json format with the follow structure  {  "cell_types": [  {  "cell_type": "",  "change": "",  "degree": "",  "change_type": "",  "value": ""  },  {  "cell_type": "",  "change": "",  "degree": "",  "change_type": "",  "value": ""  }  ]  } |
| --- |

Table S3. Aliases for cell type names. Note that misspelled aliases are present in actual data

| **Cell type** | **Aliases** |
| --- | --- |
| B cells (CD19+) | "B cell","B cells","CD19 B cell","CD19 B cells","CD19 cell","CD19+","CD19+ B cell","CD19+ B cells","CD19+ cells","Normal CD19+ B cells","CD19","CD19 cells","CD20+","CD20+ B cells","CD20+ cells","CD20","CD20 B cells" |
| CD21+ B cells (CD21+) | "CD19+ CD21+","CD19+ CD21+ B cells","CD19+CD21+ B cells","CD21","CD21 B cells","CD21 bright B cells","CD21 bright to low B cells","CD21+","CD21+ B cells","CD21+ B cells (CD19+CD21+)","CD21+ cells","CD21+ mature B cells","CD21bright B cell","CD21bright B cells","CD21bright to low B cells","mature (CD21+) B cells","mature CD19+CD21+ B cells","mature CD21+ B cells","CD21+ CD19+ B cells" |
| CD21low B cells (CD21-) | "CD19+CD21- B cells","CD21 low B cells","CD21-","CD21- (immature) B cells","CD21- B cells","CD21- cells","CD21dim B cells","CD21dim autoreactive B cells","CD21dim or CD21low","CD21dim/low","CD21dim/low B cells","CD21low B cell","CD21low B cells","CD21low cells" |
| CD4 T cells (CD3+CD4+) | "CD4 T cell","CD4 T cells","CD4+ T cell","CD4+ T cells","CD4","CD4 B cells" |
| CD45 | "CD45" |
| CD8 T cells (CD3+CD8+) | "CD8 T cell","CD8 T cells","CD8+ T cell","CD8+ T cells" |
| IgM-only memory B cells (CD27+IgD-IgM+) | "IgM only memory B cells","IgM-only memory B cell","IgM-only memory B cells","IgM only memory B cells (CD27+ IgM+ IgD-)","IgM-only B cells","IgM-only memory","IgM-only memory B cell population","IgM-only memory B cells (CD27+IgM+IgD-)","gM B cells (CD19+IgM+)" |
| NK cells (CD56/16+) | "NK","NK cell","NK cells" |
| Non-switched (or marginal zone) memory B cells (CD27+IgD+IgM+) | "CD19+ CD27+ IgM+ IgD+","MZ B cells","MZ memory B cells","Marginal zone","Marginal zone (MZ)","Marginal zone (MZ) B cell","Marginal zone (MZ) memory B cells","Marginal zone B cells","Marginal zone memory B cells (MZ)","marginal B cells","marginal zone","marginal zone (MZ) memory B cells","marginal zone B cells","marginal zone B cells (CD27+M+D+)","marginal zone B cells (MZ)","marginal zone memory B cells","marginal zone memory B cells (MZ)","non -switched memory B cells CD27+ IgM+ IgD+","non class switched memory B cells","non switched CD27+ IgM+ IgD+ memory B cells","non switched memory B cells","non switched memory B cells CD27+ IgM+ IgD+","non switched memory T cells","non-switched (marginal zone) B cell","non-switched (marginal zone) memory B cell subset","non-switched (marginal zone) memory B cells","non-switched memory (marginal zone)","non-switched memory (marginal zone) (CD27+IgM+IgD+) B cell subset","non-switched memory (marginal zone) B cell","non-switched memory (marginal zone) B cell subset","non-switched memory (marginal zone) B cells","non-switched memory (marginal zone) B cells (CD27+IgD+IgM+)","non-switched memory (marginal zone) memory B cell subset","non-switched memory B cell","non-switched memory B cell population","non-switched memory B cells","non-switched memory B cells (CD27+IgM+IgD+)","Marginal zone (CD27+M+D+)","Marginal zone B cells (CD27+M+D+)","Non switched memory B cells","Non-switched memory (marginal zone) B cells","marginal zone B cells (Cd27+M+D+)","marginal zone memory (MZ) B cells","non switched (marginal zone) memory B cells","non switched memory B cells (CD27+ IgM+ IgD+)","non switched memory B cells CD27+ IgM+IgD+","non-switched (marginal zone) B cells","non-switched memory","non-switched memory (CD27+IgM+IgD+)","non-switched memory (marginal zone) (CD27+IgM+IgD+) B cells","non-switched memory (marginal zone) B cell cells","non-switched memory (marginal zone) B cell population","non-switched memory (marginal zone) B cells (CD27+IgM+IgD+)","non-switched memory (marginal zone) subset","non-switched memory (or marginal zone) B cell subset","non-switched memory B cells (CD27+IgM-IgD-)","non-witched memory (marginal zone) B cells" |
| Plasmablasts (CD38+IgM-) | "Plasmablasts","Plasmablasts (C38+M-)","Plasmablasts (CD38++M-)","Plasmablasts (CD38+IgM-)","Plasmablasts (CD38+M-)","Plasmablasts (Cd38+M-)","plasmablasts","plasmablasts (CD19+ CD38+ IgM-)","plasmablasts (CD38++IgM-)","plasmablasts (CD38++M-)","plasmablasts (CD38+IIgM-)","plasmablasts (CD38+IgM-)","plasmablasts (CD38+M-)","plasmablasts CD19+ CD38+ IgM-","plasmablasts CD38+ IgM-","plasmablasts (CD19+CD38+IgM-)","plasmablasts (CD38+ IgM-CD19+)","plasmablasts (CD38+M-) B cells","plasmablasts CD19+CD38+ IgM-" |
| Switched-memory B cells (CD27+IgD-IgM-) | "CD19+ CD27+ IgM-IgD-","Class Switched memory","Class switched memory B cells","Class-switched memory (27+M-D-)","Class-switched memory B cells","Switched memory","Switched memory B cells","absolute class switched memory B cells (CD27+M-D-)","class switch memory B cells","class switched CD27+ IgM- IgD-","class switched memory B cells","class switched memory B cells (CD27+M-D-)","class switched memory B cells CD27+ IgM- IgD-","class-switched memory B cells","switched memory","switched memory (CD27+IgM-IgD-) B cells","switched memory B cell","switched memory B cell (CD19+ CD27+ IgM- IgD-)","switched memory B cell population","switched memory B cell subset","switched memory B cells","switched memory B cells (CD27+IgD-IgM-)","switched memory B-cells","Class switched memory B cells (CD27+M-D-)","class switched (CD27+M-D-)","class switched memory B cell subset","class switched memory B cells (CD27+ IgM- IgD-)","class switched memory B cells subset (CD19+ CD27+ IgM- IgD-)","class switched memory b cells (CD27+M-D-)","class-switched memory B cells (CD27+M-D-)","switched memory (CD27+IgD-IgM-)","switched memory B cells (CD27+ IgM- IgD-)","switched memory B cells (CD27+IgM-IgD-)","switched memory B cells CD27+ IgM- IgD-" |
| T cells (CD3+) | "CD3+ T cell","T cell","T cells","Total T cells","CD3 T cell","CD3+ T cells" |
| Total IgM+ B cells (CD19+IgM+) | "IgM B cells","IgM B cells (CD19+IgM+)","IgM+ B cells","IgM+B cells","total IgM+ B cell","total IgM+ B cells","total IgM+ B cells (CD19+IgM+)","total of IgM+ B cells (CD19+IgM+)","Total IgM+ B cells","Total memory B cells (CD27+)","total IgM-positive B cells" |
|  | "CD27+ memory B cells","Memory B cells","Total memory B cells","memory B cell","memory B cells","total B cell pool","total memory (CD27+) B cells","total memory B cell population","total memory B cells","total memory B cells ( CD27+ CD19+)","total memory B cells (CD27+)","total memory B cells CD19+CD27+","total memory CD19+ CD27+","total memory CD19+CD27+","total CD27+ memory B cells","total memory B cell (Cd27+)","total memory B cell pool","total memory B cells (CD19+CD27+)","total memory B cells (CD27+CD19+)" |
| Total memory B cells (CD27+) | "Transitional B cells","Transitional B cells (CD38+IgM+)","Transitional zone B cells","transitional B cell","transitional B cell (CD38+IgM+)","transitional B cells","transitional B cells (CD19+ CD38+ IgM+)","transitional B cells (CD38++IgM+)","transitional B cells (CD38+IgM+)","transitional B cells CD19+ CD38+ IgM+","transitional B cells CD38+ IgM+","transitional zone B cells","transitional zone B cells (CD38+ M+)","transitional zone B cells (CD38+M+)","Transitional zone B cells (CD38+M+)","transitional B cells (CD19+ CD38+ IgM+ )","transitional B cells (CD19+CD38+IgM+)","transitional B cells (CD38+ IgM+ CD19+)" |
| Unknown | "B cel subsets","B cell lymphocytosis","B cell memory subset","B cell subset","B cell subsets","CD19+/CD20+ B cells","CD19dimCD20-","CD21 expression","IgM","Memory B cell subsets","memory B cell subsets","other B cell subsets","peripheral T cells","total B cells","CD21 expression pattern","Memory B cells (CD27+)","Pan-T cells" |

Table S4. Aliases for direction of change.

| **Direction** | **Aliases** |
| --- | --- |
| Decreased | “decrease","decreased","essentially absent","negligible decrease","mild decrease","significant decrease" |
| Increased | "Increased","expansion","increase","increased","mild increase","negligible increase" |
| Unknown | "abnormal","change","Absent","dim","Insufficient data" |
| Normal | "Neither","None","absence","absent","closer to normal","neither","no change","no expansion","no significant expansion","no significant increase","none","normal","not reported","resolved","Normal","minimal" |
| Skewed | "skewed","skewing" |

Table S5. Aliases for degree of change.

| **Change type** | **Aliases** |
| --- | --- |
| Both | "both","neither","unspecified","count and frequency","count and relative frequency","percentage and absolute number" |
| Count | "Count","count" |
| Distribution | "relative distribution" |
| Unknown | "N/A","None","intensity","lymphocytosis","lymphopenia","n/a","not applicable","not reported","not specified","none" |
| Percentage | "relative","Percentage","frequency","percentage","relative frequency","relative frequency (%)" |

Table S6. Aliases for type of change.

| **Degree** | **Aliases** |
| --- | --- |
| Mild | "Mild","marginal","marginally","mild","mild to moderate","minimal","minimally","minor","small","mild-to-moderate","mildly" |
| Moderate | "moderate","moderately","modest","some","Moderate","N/A" |
| Unknown | "Neither","None","absolute","apparent","borderline","complete","inordinately","insignificant","n/a","negligible","neither","not applicable","not reported","not specified","relative","unclear","unspecified","likely","likely not clinically significant","likely total","none","proportionate" |
| Significant | "Significant","marked","profound","profoundly","severe","significant","significantly","substantial" |
